# On the relationship between the load and the variance of relative fitness

**DOI:** 10.1186/1745-6150-6-20

**Published:** 2011-04-14

**Authors:** Emmanuil E Shnol, Elena A Ermakova, Alexey S Kondrashov

**Affiliations:** 1 Institute of Mathematical Problems in Biology, Russian Academy of Sciences, Pushchino, Moscow Region, 142290, Russia; 2 Department of Bioingeneering and Bioinformatics, M. V. Lomonosov Moscow Federal University, Moscow, 119992, Russia; 3 Life Sciences Institute and Department of Ecology and Evolutionary Biology, University of Michigan, Ann Arbor, MI 48109, USA

## Abstract

**Background:**

Operation of natural selection can be characterized by a variety of quantities. Among them, variance of relative fitness *V *and load *L *are the most fundamental.

**Results:**

Among all modes of selection that produce a particular value *V *of the variance of relative fitness, the minimal value *L*_*min *_of load *L *is produced by a mode under which fitness takes only two values, 0 and some positive value, and is equal to *V/(1+V)*.

**Conclusions:**

Although it is impossible to deduce the load from knowledge of the variance of relative fitness alone, it is possible to determine the minimal load consistent with a particular variance of relative fitness. The concept of minimal load consistent with a particular biological phenomenon may be applicable to studying several aspects of natural selection.

**Reviewers:**

The manuscript was reviewed by Sergei Maslov, Alexander Gordon, and Eugene Koonin.

## Background

Let us consider the simplest model of selection, where fitness *w *∈ *[0, w*_*max*_*]*, which takes arbitrary distinct values *w*_*1*_*, ..., w*_*k *_with probabilities *p*_*1*_*, ..., p*_*k*_, (k ≥ 1 is an arbitrary number) is the only characteristic of an individual. Then, selection in the population is completely described by the probability distribution of fitness. Two functionals of this distribution, load *L *and variance of relative fitness *V*, are often used to characterize selection:(1)

and(2)

where(3)

is the mean population fitness.

Informally, *L *relates fitnesses of individuals to the maximal fitness existing within the population, and *V *relates them to the mean fitness within the population. Obviously, *L *and *V *are different characteristics of selection, in the sense that knowing one of them is usually not enough to infer the other one. However, they are not completely independent; in particular, both *L *and *V *are equal to 0 if and only if selection is absent and the probability distribution of w is concentrated at one point, so that *= w*_*max*_. Here we investigate the relationship between *L *and *V*.

## Results

*Theorem. Among probability distributions of w supported on a fixed interval [0, w_max_] (w_max _> 0) that produce a particular value of V, the minimal value of L, equal to V/(1+V), is produced by a probability distribution that consists of two atoms: 0, with probability V/(1+V), and w_max_, with probability I/(1+V)*.

Proof. Note that *L *= 1 - *J*_1_/*w*_max _and , where  is the *n*-th moment of *w*. We need to find the minimal value of *L *, and, thus, the maximal value of *J*_*1*_, consistent with a given *V*. Because , the ratio  determines *V *and *vice versa*. Because selection depends only on relative fitnesses, *L *and *V *do not change when *w *is multiplied by a positive constant *c*. Let us choose *c = 1/w*_*max *_and consider a normalized random variable *s = w/w*_*max*_. This variable takes values *s*_*j *_= *w*_*j*_/*w*_max_, confined between 0 and 1, with the same probabilities *p*_*j *_which characterize *w*. The first two moments of *s *are given by  and . Obviously,*I*_2 _≤ *I*_1_. Because , we have *I*_2_/*I*_1 _= (1+*V*)*I*_1 _.Because the right-hand side of the last equality does not exceed 1, *I*_1 _≤ 1/(1+*V*), where the equality is achieved only when *I*_1 _= *I*_2_. This is possible only when all *s*_*j *_are equal to either 0 or 1, in other words, when the sums which define *I*_1 _and *I*_2_contain only two terms. In this case, selection is extremal in the sense that, under a given *V*, *I*_1 _is maximal and, thus, *L *is minimal. Because maximal *I*_1 _is equal to *1/(1+V)*, minimal load *L*_*min *_is equal to *V/(1+V)*.

Let us now calculate the probabilities *p*_*0 *_and *p*_*1 *_with which the two values of fitness, zero and maximal, occur under extremal selection. The expectation of *s*, *I*_1 _= 0 *p*_0 _+ 1 *p*_1_. Thus, *p*_1 _= *I*_1 _and *p*_0 _= 1 - *I*_1 _. Thus, under the maximal value of *I*_1_, *p*_0 _= *V*/(1+*V*) and *p*_1 _= 1/(1+*V*).

## Discussion

We have shown that the minimal value of *L*, for a given *V*, is achieved when individuals can only have either zero fitness or a particular positive fitness *w*_*max*_. The fraction of those with zero fitness, which also equals the minimal value of *L*, is determined by *V *and is equal *to V/(1+V)*. The analogous results can be proven, using Stieltjes integrals, if *w *is a continuous random variable. Of course, there is no maximal value of load consistent with a particular *V*, because introducing an arbitrarily small fraction of individuals with very high *w *will increase *L *without affecting *V*.

The concept of (genetic) load is controversial. On the one hand, the dependency of *L *on *w*_*max*_, which may represent fitnesses of only a tiny fraction of individuals, or even describe fitness of some "ideal" individuals which are too rare to be present in any population of a realistic size, led to criticism of this concept [1]. Indeed, two populations with different values of *L *may be essentially the same, if the only difference between them is due to presence *vs*. absence of a very small fraction of individuals with a high *w*. On the other hand, *L *appears to be an important characteristic of selection, because it determines the minimal fecundity which is consistent with survival of the population. In the simplest case of an asexual population, the maximal number of offspring that an individual must be capable of producing needs to be at least *1/L*, to ensure that  ≥ 1 and, thus, that the population does not go extinct [2].

The concept of minimal load may help to resolve this controversy. If the minimal load, consistent, for example, with a particular genomic rate of deleterious mutations or a particular rate of changes of the environment, is high, this means that the population under such conditions cannot survive unless it consists of very fecund individuals. Indeed, the load determines the minimal fecundity which is still sufficient for a population to not go extinct and, when the load is minimal, for a given *V*, maximal-fecundity individuals are common in the population. Also, the concept of minimal load may be helpful for studying the properties of selection in nature. Because measuring *w*_*max *_is much harder than measuring , *L *is more difficult to measure than *V *and our result provides a possibility for estimating *L *indirectly through V, by establishing the lowest *L *consistent with an observed *V*.

Before, we considered connections between *L*, *V*, and selection differential in a more complex case of selection acting on a quantitative trait *x *[3]. Note that, according to the simplest version of Fisher's Fundamental Theorem [4], the selection differential of *w*, normalized by , is equal to *V*, because the mean population fitness after selection is *J*_*2*_/ (see [5], Chapter 3). Obviously, the situation when fitnesses of an individual can take only two values, 0 and *w*_*max *_> 0, is analogous to truncation selection in the case of selection acting on a quantitative trait other than fitness.

## Competing interests

The authors declare that they have no competing interests.

## Authors' contributions

ASK formulated the problem, and EES and EAE solved it. All authors read and approved the final manuscript.

## Reviewers' comments

### Reviewer's report 1

Sergei Maslov, Brookhaven National Laboratory, USA

This reviewer provided no comments for publication.

### Reviewer's report 2

Alexander Gordon, University of North Carolina at Charlotte, USA

Review of the paper "On the relationship between the generic load and the variance of relative fitness" by Emmanuil E. Shnol, Elena E. Ermakova, and Alexey S. Kondrashov

The authors show that among all probability distributions of fitness w supported on an interval [0,*w*_max_] with a given variance V of the relative fitness *w*/, the smallest possible value of the gene load *L *= 1 - *w*/_max _equals *V*/(1 + *V*) and is attained if and only if the fitness takes only the values 0 and w_max _with probabilities *V*/(1 + *V*) and 1/(1 + *V*), respectively. The authors do this for discrete probability distributions with finitely many values *w*_j _but mention that this restriction is not important.

The statement and the proof are correct (although quite a few changes are necessary, see "Corrections and suggestions" below). However, this result is purely mathematical in nature. Its significance for population genetics should be assessed by an expert in that field.

...

**Review of the revised paper "On the relationship between the load and the variance of relative fitness" by Emmanuil E. Shnol, Elena E. Ermakova, and Alexey S. Kondrashov**.

**A generalization**.

Here we will show how, using a different idea, the theorem can be established in its full generality - without assuming that the fitness *w *has a discrete distribution. The proof is straightforward and relies on three simple lemmas that follow the proof. The case *V *= 0 of the theorem is trivial, so we will assume that *V *> 0.

Let *w *have a probability distribution supported on [0,*w*_max_] and not concentrated at its endpoints (that is, Pr{0 <*w *<*w*_max_} > 0). We can replace this distribution by a new one that is supported on the two-point set {0,*w*_max_} and has the same mean . (By Lemma 1, such a distribution exists and is unique.) This new distribution has a strictly greater variance (Lemma 2) and consequently a strictly greater value of *V *than the original one: *V *>*V*_0_. Let us further change the new distribution by continuously increasing the mass of the atom at *w*_max _and decreasing the mass of the atom at 0 so that the total mass remains equal to 1. Then  will be strictly increasing, while both and Var *w*, and hence *V *= Var *w*/()^2^, will be changing continuously. At the end, *V *= 0. Therefore, at some intermediate point we will have *V *= *V*_0 _and a strictly increased .

This shows that, in order to maximize  (or equivalently, minimize *L*) for a given *V *> 0, it suffices to consider distributions supported on the two-point set {0,*w*_max_} and having the prescribed value of *V*. But there is exactly one such distribution (Lemma 3).This completes the proof of the theorem.

*Lemma 1*. Given *w*_0 _Є (0,*w*_max_), there exists exactly one distribution consisting of two atoms, at 0 and *w*_max_, and whose mean  equals *w*_0_.

*Proof*. Let Pr{*w *= *w*_max_} = *p*; then *w*_0 _= *pw*_max_, and we should have *p *= *w*_0_/*w*_max_.

*Lemma 2*. Suppose a random variable *w *is supported on the interval [0,*w*_max_] and has a given mean  = *w*_0 _Є (0,*w*_max_). The variance Var *w *attains its maximum over all such random variables if and only if *w *takes only two values 0 and *w*_max _(see Lemma 1).

*Proof*. Let *u *:= *w *- *w*_max_/2, so that -*w*_max_/2 ≤ u ≤ *w*_max_/2 and we want to maximize Var *w *≡ Var *u *= **E***u*^2 ^- (**E***u*)^2 ^= **E***u*^2 ^- (*w*_0 _- *w*_max_/2) ^2^. Since |*u*| ≤ *w*_max_/2, the maximum is attained if and only if |*u*| ≡ *w*_max_/2, or equivalently *w *Є {0,*w*_max_}, with probability 1.

*Lemma 3*. There exists exactly one distribution consisting of two atoms, at 0 and *w*_max_, and having a given value of *V *(*V *> 0).

*Proof*. Let Pr{*w *= *w*_max_} = *p*, so that Pr{*w *= 0} = 1 - *p*. Then *V *= Var *w*/()^2 ^= (*p*(1 - *p*) *(w*_max_)^2^)/(*pw*_max_) ^2 ^= (1 - *p*)/*p *= 1/*p *- 1, and we should have *p *= 1/(1 + *V*).

### Reviewer's report 3

Eugene V. Koonin, National Center for Biotechnology Information, NIH, USA

This very brief manuscript defines and solves an important problem in the theory of evolution. Shnol *et al*. introduce the concept of the minimal genetic load and prove that genetic load assumes the minimal value when the fitness landscape is defined in the simplest possible way, namely with only two fitness values ("fit" and "unfit" individuals). In the extremely short but constructive Discussion, the authors explain why minimal genetic load can be useful to describe population dynamics. I have a variety of questions and minor suggestions none of which invalidates the conclusions but that could be useful to address. To begin with, I think the Discussion is a bit too brief. I am not talking about any extensive elaboration but I believe it would be useful to try and give the reader some intuitive feeling WHY the condition of the minimal genetic load is what they show it is. I think this is doable and would improve the manuscript.

The more important point comes here, at the start of the Discussion: "The same results can be proven if *w *is a continuous random variable." First, it is not 100% clear to me what "the same" means in this context. That, although W is now a continuous variable, the minimal value of L is reached when and only when all individuals accumulate in just two points on the landscape? It would be best to explain. Second, "can be proven" leaves the reader with some uneasiness. If it is simple, why not give the proof for this, more general case? If it is hard, perhaps, briefly explain why and why confidence it can be proven. If simple but tedious, perhaps, make it an Appendix?

Then, I am somewhat uncertain about the exact meaning of this key statement: "Indeed, if the minimal genetic load, consistent, for example, with a particular genomic rate of deleterious mutations or a particular rate of changes of the environment, is high, this means that the population under such conditions cannot survive unless it consists of very fecund individuals". I this paper, the minimal genetic load is defined in relation to the variance of relative fitness; does the quoted statement imply that V is deterministically depends on the other variables mentioned in that sentence? I think clarification would be helpful. Finally, a couple of minor issues: I think it would be best to say in the Background section of the abstract that L and V are AMONG the most fundamental characteristics of selection.

The proof of the theorem is written in a somewhat unusual form, interspersed with comments which I think mostly distract from the logic of the proof. It would be best, I believe, to give the proof the way it is normally done, then comment.
